# Potential of Tamanu (*Calophyllum inophyllum*) Oil for Atopic Dermatitis Treatment

**DOI:** 10.1155/2021/6332867

**Published:** 2021-12-26

**Authors:** Amadeus Pribowo, Jyothsna Girish, Marsia Gustiananda, Rakrya Galih Nandhira, Pietradewi Hartrianti

**Affiliations:** ^1^Department of Biotechnology, School of Life Sciences, Indonesia International Institute for Life Sciences, Jakarta 13210, Indonesia; ^2^Department of Biomedicine, School of Life Sciences, Indonesia International Institute for Life Sciences, Jakarta 13210, Indonesia; ^3^Department of Pharmacy, School of Life Sciences, Indonesia International Institute for Life Sciences, Jakarta 13210, Indonesia

## Abstract

Tamanu oil, derived from the nut of *Calophyllum inophyllum* L., has been traditionally used to treat various skin-related ailments. In recent years, this oil is increasingly gaining popularity as researchers continue to search for novel natural alternative therapies for various skin diseases. There have been a number of *in vitro* and *in vivo* studies investigating various skin-active properties of tamanu oil, and it has been proven to have potent anti-inflammatory, antioxidant, antimicrobial, analgesic, and even wound-healing abilities. These properties make tamanu oil an especially interesting candidate for the treatment of atopic dermatitis (AD). This multifaceted disease is marked by the disruption of the skin barrier function, chronic inflammation, and skin microbiome dysbiosis with limited treatment options, which is free from adverse events and inexpensive, making it desperate for a new treatment option. In this review, we examine previous *in vitro* and *in vivo* studies on AD-relevant pharmacological properties of tamanu oil in order to evaluate the potential of tamanu oil as a novel treatment option for AD.

## 1. Introduction

**Figure 1:**
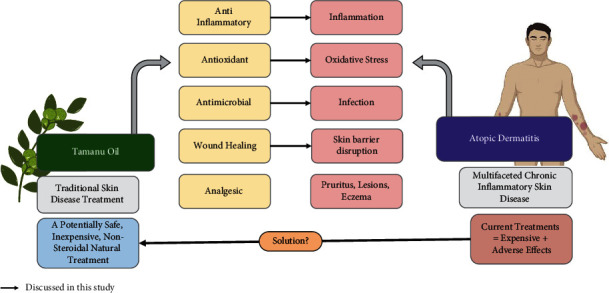
Overview of tamanu oil effects against atopic dermatitis. Tamanu oil activities (such as anti-inflammation, antioxidant, antimicrobial, and wound-healing) that are related to the pathophysiology of AD (such as inflammation, oxidative stress, infection, and skin barrier disruption) make tamanu oil a potential novel alternative treatment for AD.

Natural products have long been explored as a potential source of novel treatments, and tamanu oil is no exception. Various parts of this tree have been found to have potential use against different diseases, particularly skin-related ailments such as eczema, psoriasis, burns, acne, dermatoses, and even the treatment of wounds [1–3]. In this paper, we will review the pharmacological properties of tamanu oil and extracts derived from tamanu nuts, namely, its anti-inflammatory, antimicrobial, wound-healing, and antioxidant properties. All of these activities are expected to be beneficial to alleviate symptoms that are common in atopic dermatitis patient. Atopic dermatitis (AD) is one of the most common chronic inflammatory skin conditions of the industrialized world and has the highest disease burden among skin diseases globally [4–6]. The prevalence of AD continues to rise globally, and the literature suggests that the incidence of AD in developing countries is starting to match that in developed countries [7, 8]. While this disease affects up to 2.4% of the world's population, despite its increasing prevalence and the burden that it poses to the global public health system, both our understanding of AD and the currently available treatments for AD are still limited. AD is an extremely heterogeneous disease with a complicated etiology influenced by a combination of immunological, environmental, and genetic factors that lead to a dysregulation and dysfunction of immune responses [4]. Due to the heterogeneity of the disease, to date, AD remains incurable, and currently available therapies are limited only to symptom alleviation and prevention of AD exacerbation. A medicated topical treatment using corticosteroids is the backbone of treatment of moderate-to-severe cases [9, 10]. They are used for reducing flares and maintenance of the disease. The problem with this line of treatment is that the use of corticosteroids can have a number of both cutaneous and systemic side effects, such as skin thinning, stretch marks, and even telangiectasias [10]. Other treatments such as the use of calcineurin inhibitors, phototherapy, or systemic treatments are either expensive or come with unwanted side effects [9, 10]. Therefore, there is an urgent need for the development of nonsteroidal natural novel treatments that are not only inexpensive but also have an increased efficacy and safety profile. The various pharmacological properties of tamanu oil that have been proven in previous studies make tamanu oil a promising natural treatment to treat and ameliorate AD symptoms (as shown in Figure 1) [11]. However, there have not been any *in vitro* or *in vivo* studies that directly evaluate the use of tamanu oil for AD treatment. Hence, the purpose of this review is to extrapolate the current information based on latest studies using tamanu oil. In addition, this review will also discuss possible future directions for research in this field.

## 2. Atopic Dermatitis

Atopic dermatitis (AD) is a chronic inflammatory skin disease that can affect individuals of all ethnicities and age groups [4]. The prevalence of this disease varies greatly across countries and across age groups. AD affects approximately 20% of children worldwide [12]. In Europe, the prevalence of AD varies between 1.8% in Lithuania and 17.3% in Hungary among adolescents (13 to 14 years old) [13]. In developing countries of Asia, Africa, Latin America, and the Middle East, the incidence oscillates just as greatly as seen in the European children of the same age group, with incidence rates of 3–6% observed in the Middle Eastern regions and 12–14% in Africa. While AD predominantly affects children, adult prevalence of AD is also evident. In an international study conducted in 2018, Barbarot and his team successfully determined the adult prevalence of AD to be approximately between 2.1% and 4.9% across nations [14].

### 2.1. Diagnosis and Clinical Symptoms

AD is marked by acute flares of eczematous pruritic lesions over dry parts of the skin. The diagnosis and manifestations of AD can be subdivided into age-based profiles. Infantile AD, occurring before 2 years of age, is predominantly marked by acute eczema resulting in pruritus typically located on the head and neck. The chin, cheeks, and forehead usually exhibit these symptoms, and lesions of infantile AD are typically exudative and crusty. Lichenification may appear in these areas, secondary to scaling and redness. The extremities are also commonly affected with these symptoms, but the symptoms tend to avoid skin areas associated with the joints such as the elbows and knees. Additionally, infantile AD does not usually present symptoms in the diaper area such as the groins as hydration is generally well preserved [15].

Childhood AD progresses between the age of 2 and puberty. Unlike the infantile counterpart, AD lesions during childhood are most likely to be dry and lichenified while also classically appearing on joint surfaces such as the elbows, knees, wrists, and ankles. Childhood AD uncommonly affects the face and when it does, it mostly occurs around the mouth and eyes. Pruritus is usually more severe in children and may also be felt while sleeping. Active scratching during this age gives rise to the lichenification. In adult AD, eczema also appears in the joints with an increasing chance of it appearing in other places, such as the nipples or eyelids. The patterns formed by the affected areas are typically symmetric and dry, while the presentation of exudation is used to differentially diagnose infections from AD [15].

### 2.2. Risk Factors and Pathophysiology of AD

AD is an extremely heterogeneous disease with a complicated etiology influenced by a combination of genetic, environmental, and immunological factors that lead to a dysregulation and dysfunction of both the skin barrier function and immune responses [4]. Currently, there are two hypotheses that try to address the pathophysiology of AD. The outside-in hypothesis supports the notion that a defective skin barrier function leads to the invasion of skin and its underlying tissue by external irritants and stimuli that create an acute inflammatory response. In contrast, the inside-out hypothesis focuses on elucidating the roles of the immune system in the progression of AD [16] and proposes that the dysregulations of both the innate and adaptive immune responses give rise to AD. Recent studies seem to indicate that both skin barrier abnormalities, affected by a range of factors, and immune dysfunction contributes to the disease's pathology [4, 17]. In the following section, we present a brief review of our latest understanding on the roles of genetic, environmental, and immunological factors on the pathophysiology of AD.

#### 2.2.1. Genetic Factors

Atopic dermatitis has also been known to be caused or worsened by genetic factors. In recent years, scientists have elucidated the roles of the gene FLG, which encodes for a specific structural protein in the epidermis known as filaggrin, in AD. FLG is a gene that aids in the development of epithelial tissues [18] by strengthening the stratum corneum and acts as two of the constituents of natural moisturizing factors (NMF), which function to maintain the natural moisture barrier of the skin [19–21]. Loss-of-function (LOF) mutations of FLG gene significantly affect the integrity of epidermis and compromises skin barrier function. The inability of this barrier to function properly allows external irritants to easily attack underlying tissues and is known to increase transepidermal water loss (TEWL), contributing to the dry skin texture in AD patients (this hypothesis is termed the “outside-in” hypothesis). A number of mutations to the FLG gene have been identified in various AD cases; however, the mutations that pose the highest risk factors of AD are R501x and 2282del4 mutations. Mutations in genes other than the FLG gene have also been identified in some AD patients. Mutation on serine protease inhibitor Kazal-type 5 (SPINK5) [21] is associated with AD in Japanese populations [20]. Furthermore, protein complexes comprising the tight junction are also known to be disrupted in AD patients, resulting in an increased permeability of water, pathogens, and allergens into the skin. The roles that these mutations and other genetic predispositions may play in the pathophysiology of AD remain to be further investigated [22].

#### 2.2.2. Environmental Factors

Beyond genetic predispositions, several environmental factors are known to trigger and exacerbate AD. Environmental factors such as climate, stress, UV radiation, microbes, allergens, and pollutants can both influence the integrity of the skin barrier function and initiate an inflammatory response that triggers and exacerbates AD when an external irritant manages to gain entry through a compromised skin barrier function [23, 24]. Similar to genetic studies, our understanding on the effects of the environment on AD is still limited [7, 25, 26].

Among the environmental factors, microbes play a significant role in AD pathology. The clinical activity of this chronic inflammatory skin disease is affected by its interaction and exposure to microbes. Unfortunately, patients who have genetic defects usually face issues in the immune surveillance in the skin along with the decrease in production of antimicrobial peptides, which can increase the risk of disease. Additionally, the decrease of antigens and potentially altered pattern recognition receptors (PRRs) can result in the worsening of AD by bacteria, such as *S. aureus* and even *Malassezia furfur.* Defects present in the skin allow the entry of pathogens to the body leading to the exacerbation of the disease [27]. Recently, there has been some evidence suggesting that microbiome dysbiosis and *S. aureus* colonization can precede AD in early childhood; however, more research is required in this regard [28, 29].

The skin microbiome plays a big role in the pathology of AD, and the staphylococcal species are the main players in this factor. AD is often marked by the dysbiosis or significant decline in the diversity of the microbiome. Additionally, some studies have found that *S. aureus* seems to be one of the main colonizers of skin lesions and are linked to disease severity. These microbes have been linked to the worsening and possibly the establishment of the disease [28, 29]. Biofilm plays a major role in skin microbiota particularly in the persistence and adhesion in the cutaneous microenvironment, so it plays a large role in the function of the epidermal barrier function and therefore the local immune modulation. The immune system's chronic activation of inflammatory cytokines ends up supporting the microbe's biofilm overgrowth, and they overtake other commensal microbes changing the composition of the healthy skin microbiome. This means that targeting the microbiome can lead to a new therapeutic strategy [28–31].

#### 2.2.3. Immunological Factors

The pathophysiology of AD is affected by the myriad of immune responses occurring inside the human body. As discussed in the previous section, one of the molecular hallmarks of AD is a decrease in the production of skin antimicrobial peptides (AMPs). AMPs are a group of amphipathic proteins that are expressed predominantly by keratinocytes to neutralize toxicity derived from pathogenic bacterial species inhabiting the skin. Prime examples of AMPs are human B-defensins (hBDs) and cathelicidins. A decrease in these AMPs is positively correlated to high TEWL index, high pH, and *S. aureus* (SA) growth. Both IL-4 and IL-13, the signature cytokines of type II inflammatory response, have been shown to lead to a downregulation of several AMPs [32]. Low AMPs allow for the colonization of SA and the eventual introduction of superantigen (SA) into the skin, consequently mounting a Th-2 inflammation response. Upon the introduction of superantigen into the skin, antigen uptake is performed by resident dendritic cells (DCs) after possible activation by keratinocyte-derived IL-25, IL-33, and thymic stromal lymphopoietin (TSLP). DC activation may also be mounted by type-2 innate lymphoid cells (ILCs) via the synthesis of IL-5 and IL-13. The presentation of the antigen to T-cells and its subsequent activation marks the threshold of innate immunity [16, 33].

Once the T-cells are activated, the Th-2 cells and their associated chemokines are responsible for inducing several molecular allergy hallmarks. Notably, IL-4, IL-5, IL-10, IL-13, IL-33, and CCL31 are able to promote B-cells class-switch to IgE, inhibit filaggrin, and downregulate AMPs even more. Indeed, another molecular hallmark of AD is the heightened levels of IgE, which subsequently induces the manifestation of observed symptoms (REF). Th-2 response will eventually cease its activities, and recent studies have shown that in later stages of AD, Th-1 response is increasingly favoured. This gives rise to increased IFN-*γ* levels and induces apoptotic signals on keratinocytes, which further disrupted the skin's barrier function. Increased levels of IL-17 and IL-22 are observed in AD lesions, with the latter triggering epidermal hyperplasia and termination of keratinocyte differentiation; at this stage, adaptive immunity takes center stage [16, 33].

#### 2.2.4. Complications with Current Atopic Dermatitis Treatments

The multifaceted nature of AD makes the treatment of the disease tricky. AD patients usually have a combination of both skin inflammation and skin barrier dysfunction, meaning that a good therapeutic agent should aid in the solving the inflammatory response as well as the repair of the barrier [34]. A number of AD therapies exist today. These therapies include nonmedicated as well as medicated topical treatments using corticosteroids and calcineurin inhibitors, phototherapy, and a number of systemic treatments, such as oral corticosteroids, azathioprine, methotrexate, and cyclosporine A [10]. The first line of treatments for mild AD typically includes using nonmedicated emollients to ease the dry and cracking skin and reduce the appearance of AD. Calcineurin inhibitors such as Pimecrolimus can also be applied as it is able to overstep some of the side effects of corticosteroids, such as skin thinning. While topical corticosteroids work by activating our nuclear glucocorticoid receptors, thereby changing cytokine expression, topical calcineurin inhibitors disrupt the early T-cell activation and the following release of cytokines. While calcineurin inhibitors are considered safer options, this treatment comes with its own set of concerns including erythema, pruritus, transient burning, and also a boxed warning about potential carcinogenesis associated with long-term use [9]. Therefore, the arsenal of topical treatments is desperate for a safer addition [10]. In addition, the systemic treatment of AD is both limited and difficult with dupilumab being the only approved biologic used for moderate and severe cases of AD in children aged 6 and older as this biologic is the only one with a consistent long-term efficacy and even safety trial data [4].

Apart from having limited options for treatment, AD patients also incur a significant economic burden upon the diagnosis of this disease. Unfortunately, not all patients are able to manage this disease with simple topical treatments. As the disease progresses, patients typically require an increase in the intensity of the treatment or the use of different treatments concurrently. This drives their cost of treatment up significantly. Typically, patients start with emollients then move to either corticosteroids or calcineurin inhibitors. If these fail, they move on to more advanced treatments such as the aforementioned phototherapy option or even start using systemic immunosuppressant. These immunosuppressants can include cyclosporin, methotrexate, and others. A study conducted by Eichenfield et al., published in 2020, evaluated the costs as well as treatment patterns of patients suffering from AD in the United States. This study found that the mean per-patient healthcare cost for patients with moderate-to-severe forms of the disease can be up to $20,722/year. Most patients require a switch of medication or an additional form of treatment due to the unwanted effects of current therapies [35]. This study worked on highlighting another reason compelling scientists to search for safe and inexpensive form of treatment for AD, such as tamanu oil.

## 3. Tamanu Oil

### 3.1. What Is Tamanu Oil?

**Figure 2:**
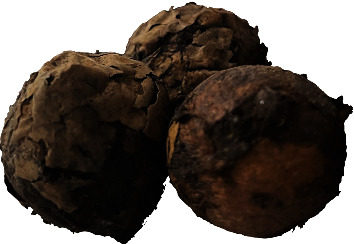
Tamanu nut. Dry tamanu nuts prior to extraction of the oil or extract. The shell of the nut is typically removed to reveal the white kernels from which oil is typically extracted.

Tamanu oil, derived from the nuts of *Calophyllum inophyllum* L., is obtained from an evergreen, pantropical tree growing around seashores in Asia, Africa and Pacific countries. In French Polynesia, the tree is locally called “tamanu” tree, and various parts of the plant have been used over time for various medicinal purposes and even as active ingredients in cosmetics [2]. One of the most commonly used parts of the plant is the nut as depicted in Figure 2. The kernels have an oil content of around 75%. The oil obtained from the nuts of the tamanu plant has been traditionally topically applied on the skin and mucous membrane lesions to prevent skin infections and even to reduce the appearance of a scar. Over the years, tamanu oil has been recommended for various skin issues such as eczema, acne, psoriasis, burns, skin cracks, and dermatoses. It has also been used for its pain-relieving properties in rheumatisms and sciatica and for its analgesic properties in wound healing [1, 2]. In addition, tamanu oil is used in Fiji to treat a number of other ailments, such as joint pain and conjunctivitis, and even to prevent rash in infants.

These traditional uses of tamanu oil have attracted the attention of the scientific community. A number of studies have tried to identify different pharmacological properties of the oil and nut extracts [36–38]. A study conducted by Yimdjo et al. in 2004 revealed some antimicrobial properties of the oil, while other studies found other biological properties such as wound-healing, antifungal, and anti-inflammatory properties [39]. This review will examine these properties in relation to the pathophysiology of AD and to provide possible future directions for studies exploring the possibility of using tamanu oil for this disease.

### 3.2. Pharmacological Properties of Tamanu Extract Related to AD

#### 3.2.1. Anti-Inflammation

Skin barrier dysfunction in AD facilitates entry of the allergens and infectious agents into the skin, which induces immune responses that lead to inflammation [40]. Inflammation is a mechanism of the host defense to eliminate the pathogens and as a preceding step for the ensuing process of tissue repair [41]. However, a prolonged inflammatory process such as one that occurs in AD will disrupt immune homeostasis and inhibit wound repair significantly, creating a vicious cycle of inflammation [42]. A number of studies have elucidated the anti-inflammatory property of tamanu oil both *in vitro* and *in vivo*, which makes this oil a promising candidate for AD treatment. While studies have also showed that other parts of the tamanu plants also have anti-inflammatory activity [43–45], this review paper will solely focus on the anti-inflammatory properties of tamanu oil and extracts obtained from tamanu nuts.

In an *in vitro* study, tamanu oil extract has shown to be able to inhibit two key enzymes involved in inflammation, namely, proteinase K (PTA) and lipid oxygenase (15-LOX) [46]. LOX is the enzyme that catalyzes the oxidation of arachidonic acids to yield leukotrienes that mediate inflammation and have been implicated in AD [46, 47]. Proteinase K is a serine protease enzyme. Aberrant epidermal serine protease activity is implicated in the pathogenesis of AD not only in lesions but also in nonlesions of AD patients [48]. Cassien et al. tested several different fractions of the ethanol-soluble resinous component of tamanu oil (EtTO) and its subsequent neutral (NTR) and acidic (ATR) fractions preparations. ATR, NTR, and EtTO extracts were shown to be able to inhibit 62–72% of PTA activity, which was nearly equal to the inhibition by standard drug diclofenac (64%) when tested at the same concentration of 150 *μ*g/mL. NTR and ATR inhibit 45–60% of 15-LOX activity, while standard compound quercetin inhibits 83% when tested at the same concentration of 100 *μ*g/mL [46].

The anti-inflammatory properties of tamanu oil, extracts, and/or their constituents have also been demonstrated *in vivo*. Calophyllolide (CP), one of the active compounds in tamanu oil, was shown to reduce the capillary permeability in mice when induced by various chemical mediators involved in the inflammatory process such as histamine (HA), 5-hydroxytryptamine (5-HT), and bradykinin (BK), and it has a similar safety margin with the standard anti-inflammatory drug oxyphenbutazone [49]. During an inflammation, HA, HT, and BK are able to increase vascular permeability that allow fluid and immune cells to enter the tissue and induce inflammation [50, 51]. The anti-inflammatory property of CP was also reported by Nguyen et al., who observed a marked reduction in myeloperoxidase (MPO) activity on the wound skin tissue samples of mice upon treatment with the CP. MPO is an enzyme that catalyzes the formation of reactive oxygen intermediates, a hallmark of inflammation [52]. In addition, this study also demonstrated that CP treatment prevented a prolonged inflammatory process by downregulating the systemic proinflammatory cytokines such as IL-1*β*, IL-6, and TNF-*α* and by upregulating the expression of the anti-inflammatory cytokine IL-10. Furthermore, CP contributes to wound-healing activity by inducing the switch of macrophages from the M1 into M2 phenotype. CP treatment to the wound area resulted in the downregulation of genes related to the macrophage M1 phenotype (CD14 and CD127) and upregulation of the M2-related genes (CD163 and CD206) when compared to the vehicle [52]. While the M1 macrophage is inflammatory and contributes to the elimination of microbial pathogens at the site of the wound, M2 macrophage phagocytes damaged cells at the site of injury, which is also beneficial for the wound-healing process [52].

Despite this mounting evidence of tamanu oil having anti-inflammatory compounds, to date, there is no study that directly tests tamanu oil and its constituents for AD-related skin inflammation. The availability of a cell-based model of AD such as HaCaT cells induced by the combination of cytokines implicated in the AD condition (TNF-*α*/IFN-*γ* or IL-4/IL-13) [53] may pave the way to a more detailed study on the mechanism of action of tamanu oil to ameliorate AD symptoms.

#### 3.2.2. Antimicrobial Activity

Tamanu oil potentially contributes to enhancing the protection of the skin barrier function against microbial assaults through at least 2 antimicrobial mechanisms: direct inhibition of microbial growth and stimulation or modulation of skin immunity. Both the oil and the extract of tamanu nuts have been shown to have antimicrobial activities. Tamanu oil has been shown using oilogramme to directly inhibit the growth of Gram + bacteria implicated in skin infection such as *Staphylococcus aureus, Bacillus cereus, Staphylococcus epidermidis, Staphylococcus haemolyticus*, and *Corynebacterium minutissimum* as well as those implicated in acne *Propionibacterium acnes* and *Propionibacterium granulosum* with a minimal inhibitory concentration (MIC) value that ranges from 0.01 to 0.5%. This range of MIC value was shown to be similar or lower than that of ofloxacin. Using a technique called bioautography in which the components in tamanu oil were separated based on their polarity using Thin-Layer Chromatography (TLC), the authors showed that it was the resin fraction of the oil, and not its fatty acids, that was responsible for the observed antimicrobial activity against *S. aureus* [37]. Another study showed that compounds isolated from the crude extract of *Tamanu* nut following CH2Cl2 : MeOH (1 : 1) extraction exhibit antimicrobial activities against *S. aureus* (ATCC6538). These compounds were identified as CP (MIC: 16 *μ*g), inophyllum C (MIC: 10 *μ*g), and inophyllum E (MIC: 13 *μ*g). However, their antimicrobial activity was still lower than that of the positive control oxacillin (MIC: 30 *μ*g) [39]. In addition, ethanol extract of tamanu oil has been shown to exhibit antifungal activity against *Candida albicans, Candida tropicalis, Aspergillus niger, Aspergillus fumigatus,* and *Aspergillus tenuissima* at a concentration of 4 *μ*g/ml compared to the positive control fluconazole at 10 *μ*g/ml [38]. Interestingly, neither tamanu oil or tamanu nut extract has been shown to significantly inhibit the growth of Gram-bacteria, and to date, the mechanisms by which these compounds inhibit microbial growth remain poorly understood.

In addition to directly inhibiting microbial growth, tamanu oil also affects microbial growth by modulation of skin immunity. Tamanu oil has been shown to induce U937 derivative macrophages to release *β*-defensin 2, an antimicrobial peptide that is active against Gram-bacteria and is implicated in skin immunity [37]. The roles of tamanu oil in stimulating or modulating the skin's innate immune response warrant further research. Furthermore, as the formation of *S. aureus* biofilm has been shown to be a primary mechanism behind the pathogenesis of chronic AD [30], it remains to be determined if tamanu oil or extract could play a role in the formation of *S. aureus* biofilm.

#### 3.2.3. Wound-Healing Activity

Several studies showed that wound healing was impaired in AD, as shown by Beken et al., in 2020, where HaCaT induced with AD-stimulating agents showed delayed wound healing [54]. The study showed that AD-stimulating agents downregulated expression of E-cadherin and occludin genes, which are responsible for the production of epithelial junctional proteins. Additionally, AD-stimulating agents also upregulate the expression of MMP1, MMP2, and MMP9 genes, which are responsible for extracellular matrix (ECM) degradation. The expression of these genes is necessarily slightly increased in wound healing to facilitate cell migration. However, overexpression of these genes could actually interfere with tissue repair since it will result in excessive ECM degradation in the tissue regeneration stage [54]. Another study by Zhao et al. also showed delayed wound healing in IL-4 Tg mice model for AD despite the robust keratinocyte proliferation specific to AD pathophysiology [55].

CP was proven to show potential in supporting and accelerating wound healing based on evidence from past studies. A study by Nguyen et al., in 2017, showed that CP significantly improved wound closure area compared to vehicle (PBS) and control (Povidone-Iodine). The histological result also showed mice treated with CP displayed reduced fibrosis and also faster wound closure rate compared to vehicle and control. Interestingly, the study showed that during the proliferation stage of wound healing in the CP-treated group, the Masson-Trichrome stained section exhibited complete reconstruction of the collagen deposited on the dermal layer as well as smaller collagenous scar compared to the control group. As on the remodelling stages, it was observed that soluble collagen content was up to twice higher on CP-treated group on day 10 and reduced on day 14 when compared to control [52].

Leguillier et al. in 2015 and Ansel et al. in 2016 performed *in vitro* studies that evaluated the effects of tamanu oil or its extracts on keratinocytes as well as on keratinocytes and fibroblasts, respectively. Leguillier revealed that treatment with tamanu oil improved wound closure rate of keratinocytes represented through scratch assay up to 2.1 times compared to control, signifying its potential to be used as an active compound that may play a role in the proliferation stage of wound healing. Meanwhile, a study by Ansel showed that tamanu extracts induced glycosaminoglycans (GAG) production up to 350% compared to control on HaCaT cells. Additionally, tamanu treatments also improved collagen production of fibroblasts up to 40% compared to controls after 24 h treatment with tamanu extracts. The increase of the ECM production proved that tamanu treatment showed potential to be beneficial in the proliferation stage of wound healing. This was also further confirmed by Ansel through the scratch assay performed on human dermal fibroblasts cells where wound closure was faster on tamanu-treated cells compared to controls and vitamin C-treated groups. Cells treated with tamanu and vitamin C and nontreated cells showed wound closure in 14, 15, and 16 h, respectively [37, 56].

Based on these findings, even though there has not been a clear mechanism on the improved wound closure of cells or animals treated with tamanu, the increased GAG, collagen production, and faster wound closure rate on keratinocytes showed promising potential for tamanu oil and/or extracts to be used in AD treatment. As AD pathology is primarily related to skin barrier dysfunction, improvement in keratinocyte proliferation and ECM production is expected to lead to improved skin barrier, assisting in the treatment of AD main pathogenesis [57].

#### 3.2.4. Antioxidant Activity

Oxidative stress may play a role in the pathogenesis of AD. Oxidative stress upregulates the expression of proinflammatory cytokines genes, which lead to inflammation. The inflammatory cells in turn release free radicals when activated [58]. Chronic skin inflammation that occurs in AD patients is associated with the overproduction of reactive oxygen species (ROS), such as superoxide (O2-) and hydrogen peroxide (H2O2) [59]. Normally, cells can neutralize ROS by using enzymatic and nonenzymatic antioxidants. However, in AD patients, the level of these enzymatic and nonenzymatic antioxidants is reported to be decreased, making AD patients more prone to damage caused by ROS or oxidants than non-AD individuals [60]. In addition, several biomarkers of oxidative stress have been shown to be elevated in AD patients as compared to non-AD individuals. Nitric oxide (NO) and malondialdehyde (MDA), both being the end products of lipid peroxidation, were observed to be significantly higher in AD patients compared to control [60, 61].

Tamanu oil has been found to have antioxidant properties due to its capability of reducing the intracellular ROS production [62]. One of the enzymes responsible for ROS production is xanthine oxidase (XO). A recent report showed that resinous component of tamanu oil and its fractions inhibit the activity of XO [46]. The authors suggested that the percentage of XO inhibition by the resin and their fractions is attributed to the presence of total phenolic content (TPC). The best XO inhibition was exhibited by ATR fraction with IC50 of 71 ± 6 *μ*g/mL. The XO inhibition by ATR is less efficacious compared to the standard polyphenol compound quercetin with IC50 of 1.52 ± 0.12 *μ*g/mL while being nearly comparable to gallic acid with IC50 of 41 ± 1.2 *μ*g/mL. Of note is the fact that deresinated tamanu oil (DTO) does not inhibit XO activity. The XO inhibitory activity of tamanu oil extract and its resinous fractions is correlated with their superoxide radical quenching property as reflected in IC50 values ranging from 14 to 25 *μ*g/mL. The metal chelating capacity (MCC) reflects the ability of the compound to inhibit iron-induced damage by forming a complex with Fe^2+^. NTR and ATR, due to the high phenolic and flavonoid content, exhibit the MCC level comparable to that of quercetin. The antioxidant property of the resinous fractions of tamanu oil was also shown by the reduced level of MDA in the 3T3 fibroblasts cells treated with *t*-butanol. At 15 *μ*g/mL, the resin was able to reduce MDA-TBA level better than quercetin at 3 *μ*g/mL. Overall, when compared to the crude tamanu oil material, the resinous fractions were shown to have significantly improved antioxidant activities [46, 62]. Taken together, these antioxidant properties of tamanu oil may potentially ameliorate AD symptoms, and hence the possibility of using tamanu oil as a potential treatment for AD warrants further investigation. Since the skin barrier defect, infection, inflammation, and oxidative stress are interrelated in AD, a multimodality therapy is needed. Tamanu has been shown to have properties, such as anti-inflammation, antioxidant, and antimicrobial, and improve skin barrier function, and hence, it can be an alternative natural product for AD.

## 4. Conclusions and Future Perspectives

AD is a complex and multifaceted disease with limited safe and inexpensive treatments available. Several studies have been conducted to investigate the anti-inflammatory, antimicrobial, wound-healing, and antioxidant effects of tamanu oil, making it a promising candidate for AD treatment. The inexpensive extraction process and natural origin of this potential treatment makes it an attractive remedy to investigate. In addition, due to the complex nature of this disease, a treatment with multiple activities such as tamanu oil is desirable. However, to truly find a suitable treatment, more research should be conducted to identify the differences between severity levels of AD so that appropriate treatments can be discovered [63]. While there is a lot of evidence supporting the possible use of tamanu oil to treat AD, to truly test this potential, appropriate *in vitro* and *in vivo* models of AD need to be developed. Active compounds responsible for the effect need to be identified and their mechanism of action needs to be elucidated. *In vitro* studies using keratinocytes induced with TFN-*α* and IFN-*γ* to model AD condition could be employed to study the anti-inflammatory and skin barrier repair abilities of tamanu oil [64]. Meanwhile, *in vivo* study that represents AD model such as transgenic mice or mice induced with allergens could also be employed to evaluate the tamanu oil activity against AD [65]. In addition, a number of bioactive compounds such as neoflavanoids tamanolide D and P, inophyllum (C, D, E, and P), calophyllolide (CP), calanolide Gut 70, and also calanolides A, B, and D have been identified in the ethanol-soluble fraction of tamanu oil [64, 65]. Investigating the properties and mechanism of action of these individual bioactive compounds in relations to AD is also needed in order to identify compounds that could potentially be developed further into a specific AD treatment agent. Filling in these gaps can contribute to the identification of which severity level of AD this treatment can be applied to, and this can also conclude whether tamanu oil can be a prospective new drug candidate for AD treatment.

## Data Availability

The data supporting this review are from previously reported studies and datasets, which have been cited. No primary data were used to support this review.
